# Psychoeducation for the Frontline: Ealing Liaison Psychiatry Service (ELPS) pilot training day for London Ambulance Service (LAS)

**DOI:** 10.1192/bjo.2021.416

**Published:** 2021-06-18

**Authors:** Emma McLean, Mariam Alexander

**Affiliations:** 1 Imperial College Healthcare NHS Trust; 2 Ealing Liaison Psychiatry Service, West London NHS Trust

## Abstract

**Aims:**

To host the first ELPS training day specifically for LAS staff to improve their knowledge and understanding about mental health issues and the role of ELPS.

On average 13,000 calls are received by LAS relating to mental health issues every month. Many patients seen by ELPS will have multiple interactions with LAS. ELPS has previously held training for the Emergency Department team but this innovative day was designed to extend this training commitment to pre-hospital clinicians

**Method:**

LAS training needs were initially assessed by a bespoke questionnaire and ELPS attending another LAS training event held by the new mental health joint response car team.

We then developed a training programme to match the identified training needs and which utilised the specific expertise of individual ELPS staff.

14 members of the local LAS stations attended including both Paramedics and Emergency Ambulance Clinicians. The presentations covered mental state examination, suicide, risk assessment, substance misuse, legal frameworks and then a ‘challenging cases’ session to bring it all together.

Pre and post course questionnaires were completed by participants, exploring attitudes and knowledge.

**Result:**

There was a statistically significant improvement in the average self-ratings for all of the categories assessed including attitudes to mental health, confidence in assessment and knowledge relating to the process the patient will experience in the emergency department.

The knowledge about the pathway and role of liaison psychiatry showed the greatest improvement with an average 4.25 increase in pre and post course rating.

Almost all participants (9.2/10) would recommend this training day to a colleague

**Conclusion:**

We met our objective of improving LAS staff knowledge and understanding about mental health issues and the role of ELPS. We plan to build on this successful pilot and expand our training programme for LAS with the ultimate aim of improving patient care.

